# Ultrahigh Permittivity of Surface-State-Dominated Bi_2_Te_3_ Nanosheets for Low-Frequency Microwave Absorption

**DOI:** 10.34133/research.0886

**Published:** 2025-10-22

**Authors:** Dengchen Li, Qiji Ma, Chengyou Lin, Chen-Ming Liang, Ling Huang, Yuhang Qi, Jianhua Li, Pei-Yan Zhao, Zhi-Ling Hou, Dongfeng Zhang, Guang-Sheng Wang

**Affiliations:** ^1^ School of Chemistry, Beihang University, Beijing 100191, China.; ^2^ CH UAV Science & Technology Co. Ltd., Taizhou 318000, China.; ^3^ College of Mathematics and Physics, Beijing University of Chemical Technology, Beijing 100029, China.; ^4^ School of Physics and Optoelectronic Engineering, Beijing University of Technology, Beijing 100124, China.

## Abstract

Low-frequency microwave absorption poses an important challenge due to the excessive thickness of absorbers, which inherently correlates with wavelength. Herein, Bi_2_Te_3_ nanosheets were synthesized via a solvothermal method to achieve high permittivity, aiming to address the issue of low-frequency microwave absorption. The as-prepared nanosheets demonstrate a tunable ultra-high permittivity (up to 282) at 2 GHz, accompanied by a loss tangent below 0.81 and pronounced frequency dispersion characteristics. The nanosheet material demonstrates a synergistic balance of ultrahigh permittivity and appropriate dielectric loss, attributed to its surface-state-conducting and bulk-insulating character. At 2.4 GHz, the composite material composed of Bi_2_Te_3_ nanosheet achieves an effective absorption with an electrical thickness of 0.032, which is significantly thinner than those reported for state-of-the-art microwave absorbing materials. By leveraging the high permittivity and pronounced frequency dispersion characteristics of Bi_2_Te_3_ nanosheet composites, broadband microwave absorption is achieved across both 2- to 6-GHz and 6- to 18-GHz ranges through multilayered architectures. This work provides a strategic approach to overcome the longstanding challenge of broadband low-frequency microwave absorption.

## Introduction

Microwave absorbing materials play a pivotal role in addressing 2 critical needs, including ensuring electromagnetic security in military stealth [1–4] and safeguarding civilian electronics from electromagnetic interference risks [5,6]. The proliferation of 5G-enabled devices has intensified electromagnetic pollution, so it is urgent to develop low-frequency (S band) electromagnetic wave absorbing (EMWA) materials [7,8]. However, limited by ^1^/_4_-wavelength resonance [9,10], traditional EMWA materials have excessive matching thickness at low frequencies, hindering their practical applications [11,12]. The key to reduce the thickness of absorbers lies in increasing the refractive index of materials, which is the real part of the square root of the product of complex permittivity and complex permeability [13]. Accordingly, there are primarily 2 strategies to decrease the thickness of absorbers. The first strategy is to increase the permeability of microwave absorbing composites by introducing magnetic materials. For example, Kong and colleagues [14] prepared a novel shape anisotropy chain-like CoNi, achieving an absorber thickness of only 0.041*λ* owing to high permeability ($\mu^{\prime }=2$) at 3.16 GHz. Hou and colleagues [15] constructed vapor-grown carbon fiber and flaky carbonyl iron particles (VGCF/CIP) composite with a high permeability ($\mu^{\prime }=2.7$), which obtained an absorber thickness of 0.035*λ* at 3 GHz. The second strategy is to increase the permittivity of composites by increasing the filling ratio of conductive absorbents. However, the high-concentration conductive fillers in EMWA materials invariably induce excessive dielectric loss, resulting in impedance mismatch between absorber and free space [16,17]. Therefore, the development of EMWA materials that combine ultra-high permittivity and appropriate dielectric loss is urgent but challenging for low-frequency microwave absorption.

Considering the unique advantage in adjustable electrical transport of topological insulator bismuth telluride (Bi_2_Te_3_) with bulk insulating and surface conducting [18,19], in this work, we prepared Bi_2_Te_3_ nanosheets via a solvothermal method. The as-prepared composite material exhibits ultrahigh permittivity and suitable dielectric loss, which attributed to the micro-capacitor effect arising from unique surface-state confinement. Based on Bi_2_Te_3_ nanosheet composites with strong frequency dispersion of permittivity, multilayer structures were designed to achieve effective absorption covering 2 to 6 GHz and 6 to 18 GHz, respectively. The mechanism of broadband microwave absorption at low frequency is revealed, providing important guidance for the field of electromagnetic wave absorption.

## Results and Discussion

**Fig. 1.**
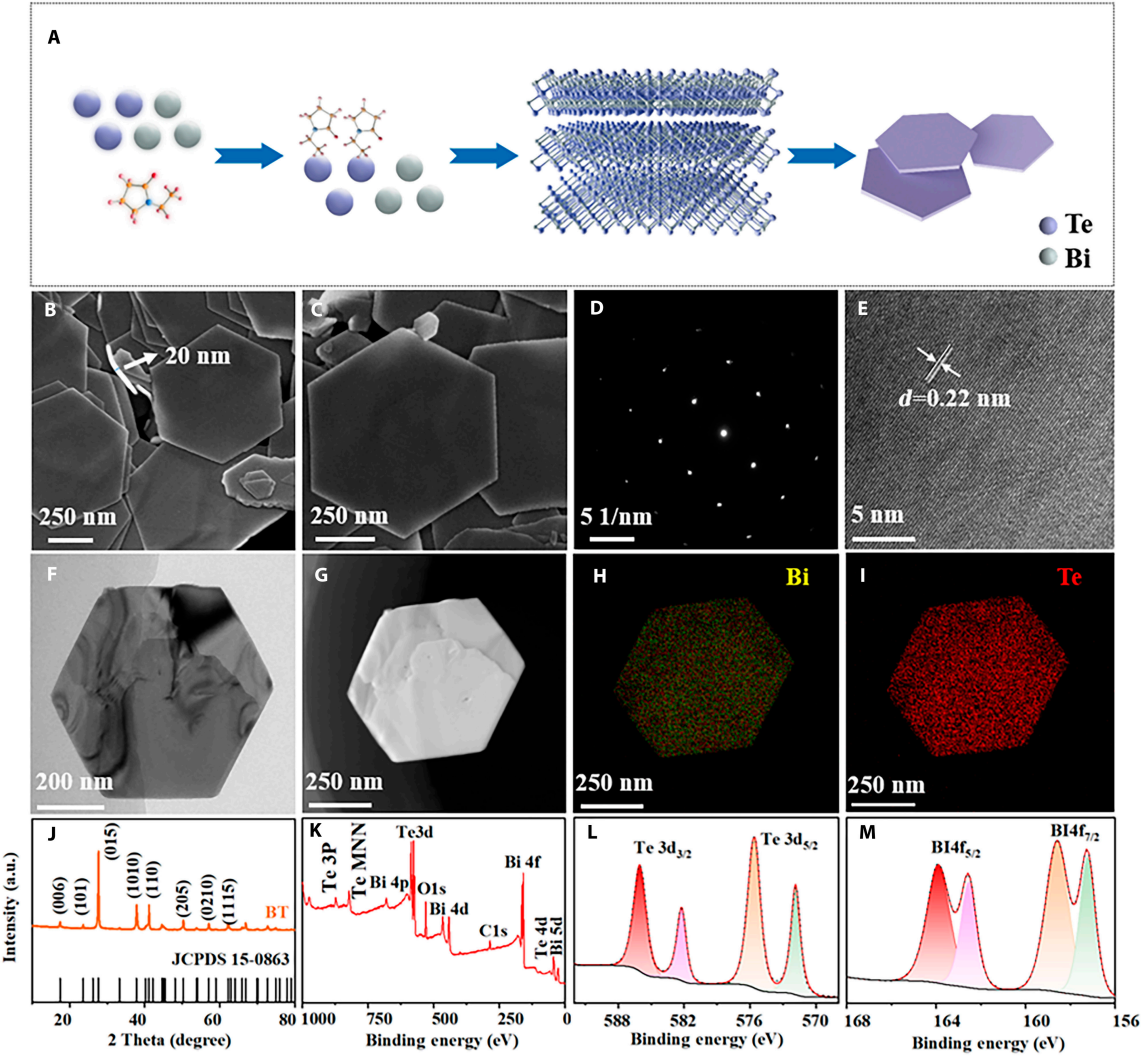
(A) Schematic of Bi_2_Te_3_ nanosheet formation. (B and C) SEM, (D) SAED, (E) HRTEM, (F) TEM, and (G to I) energy-dispersive x-ray spectroscopy (EDS) mapping of Bi_2_Te_3_ nanosheets. (J) XRD, (K) XPS survey spectrum, (L) Te 3d XPS spectrum, and (M) Bi 4f XPS spectrum of Bi_2_Te_3_ nanosheets.

Figure 1A shows the schematic of the formation of Bi_2_Te_3_ nanosheets. Bi_2_Te_3_ nanosheets exhibit a distinctive layered structure with atomic arrangement following a Te–Bi–Te–Bi–Te stacking sequence [20,21]. The layered structure shows weak Te–Te interlayer coupling but strong in-plane covalent bonding, which demonstrates higher planar structural stability [22]. Furthermore, the introduced PVP coordinates with Bi^3+^ ions along the direction perpendicular to the basal planes during the synthesis of Bi_2_Te_3_ nanosheets. This coordination suppresses the out-of-plane growth kinetics while preferentially facilitating in-plane expansion, ultimately obtaining ultrathin nanosheets with smooth surfaces. Scanning electron microscopy (SEM) images (Fig. 1B and C and Fig. S1) show that Bi_2_Te_3_ nanosheets possess a hexagonal structure with a thickness of approximately 20 nm. Selected-area electron diffraction (SAED) image of Bi_2_Te_3_ nanosheets (Fig. 1D) shows distinct hexagonal symmetry spots, further confirming the high crystallinity and single-crystalline characteristic of the nanosheets. High-resolution transmission electron microscopy (HRTEM) image as shown in Fig. 1E confirms the crystallographic orientation, with the interplanar spacing of 0.22 nm corresponding to (110) plane of Bi_2_Te_3_. Bi_2_Te_3_ nanosheet (Fig. 1F) maintains a well-defined hexagonal morphology, which is in good agreement with SEM results. Concurrently, wavelike contrast variations are observed on the nanosheet surfaces, which can be attributed to either surface strain or thickness inhomogeneity. Figure 1G to I shows that the homogeneous nanosheets consist exclusively of Bi and Te elements, with no detectable impurity phases present. Figure 1J presents the x-ray diffractometer (XRD) patterns of the material. It is observed that the diffraction peaks of samples are all well-matched with the standard card (PDF#15-0863), confirming the single-phase Bi_2_Te_3_ crystal structure (space group: $R\bar{3}m$). Moreover, the high intensity of the diffraction peaks further demonstrates the good crystallinity of the sample. Figure 1K confirms that as-prepared nanosheets are predominantly composed of Bi and Te elements [23,24]. As shown in Fig. 1L, the x-ray photoelectron spectroscopy (XPS) spectrum of Te3d exhibits 2 peaks at 571.7 and 582.1 eV, corresponding to Te3d_5/2_ and Te3d_3/2_, respectively [25]. Two additional peaks at 575.5 and 586.0 eV are also observed, corresponding to the Te3d spectrum of Bi_2_Te_3_ in its oxidized state, respectively. Figure 1M shows the XPS spectrum of Bi 4f, with peaks at 157.3 and 162.8 eV corresponding to Bi 4f_7/2_ and Bi 4f_5/2_ [26], while peaks at 158.5 and 164.1 eV are associated with the oxidized state of Bi in Bi_2_Te_3_. These findings confirm the chemical states of both Te and Bi in the Bi_2_Te_3_ nanosheets.

**Fig. 2.**
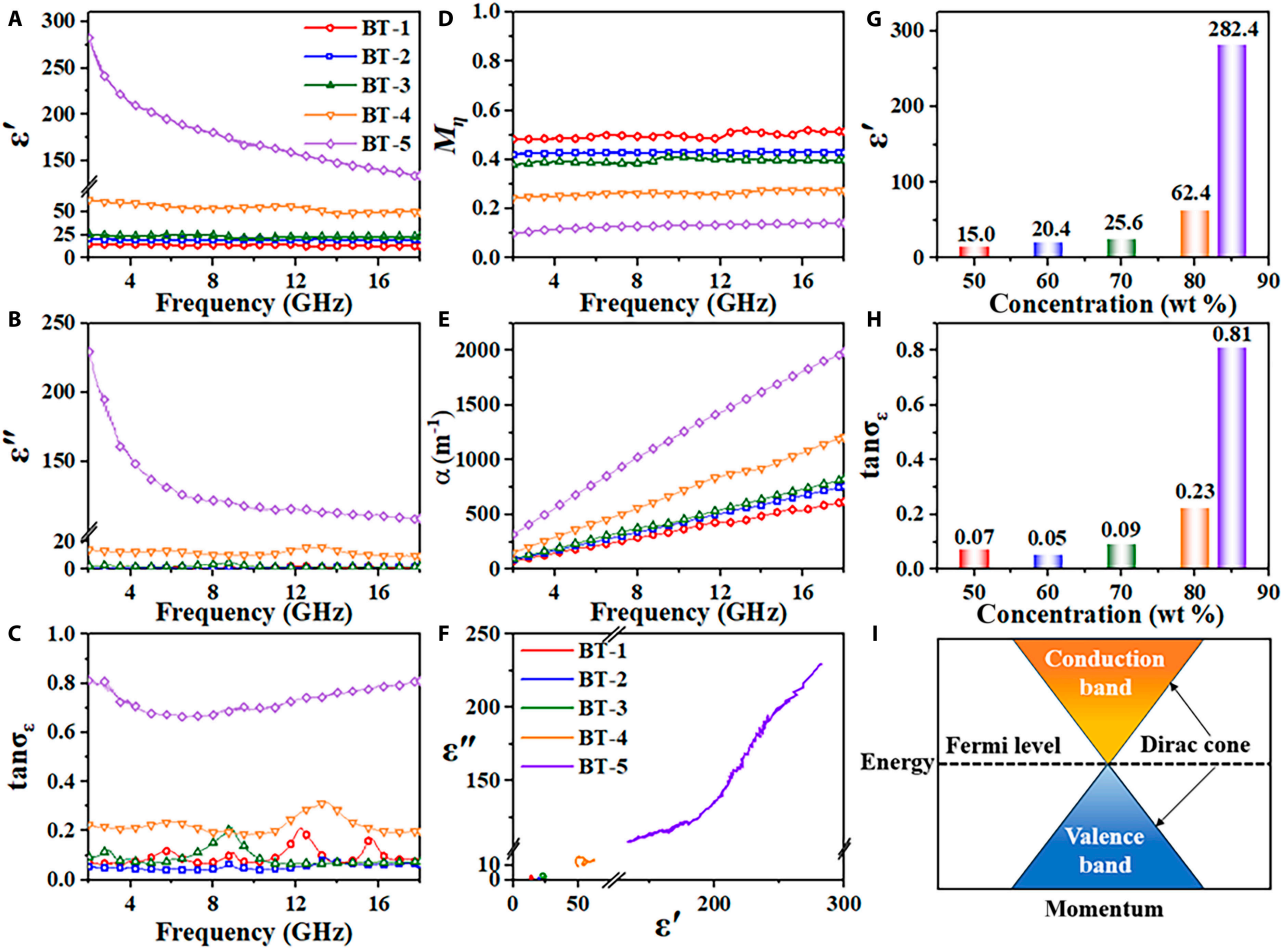
Frequency dependence of (A) $\varepsilon^{\prime }$, (B) $\varepsilon^{\prime \prime }$, and (C) dielectric loss tangent. (D) Wave impedance matching coefficient, (E) attenuation coefficient, and (F) Cole–Cole curves. Dependence of (G) $\varepsilon^{\prime }$ and (H) dielectric loss tangent with filling ratios of Bi_2_Te_3_ nanosheets at 2 GHz. (I) Schematic of energy band structure.

Figure 2A and B shows frequency dependences of $\varepsilon^{\prime }$ and $\varepsilon^{\prime \prime }$ for BT-1, BT-2, BT-3, BT-4, and BT-5 samples, respectively. As the frequency increases, both $\varepsilon^{\prime }$ and $\varepsilon^{\prime \prime }$ show a fluctuating downward trend, demonstrating a Debye relaxation [27,28]. Furthermore, both $\;\varepsilon^{\prime }\;$and $\varepsilon^{\prime \prime }$ escalate with increasing filling ratio of Bi_2_Te_3_ at the same frequency. Particularly at a filler loading of 85 wt %, this composite exhibit pronounced dielectric dispersion, making it a preferred candidate material for multilayer broadband absorbing patch design. The permittivity of Bi_2_Te_3_ nanosheet composites can be flexibly adjusted from 5 to 282 at 2 GHz by changing the filling ratio. The permittivity of Bi_2_Te_3_ nanosheet composite reaches 282 while maintaining a dielectric loss tangent below 0.81 (Fig. 2C), which is attributed to the micro-capacitor effect caused by its unique surface-state confinement. For other conductive composite materials such as graphene [29] and carbon nanotubes [30] when the permittivity exceeds 60, the dielectric loss will be much higher than the permittivity, which leads to impedance mismatch between the air and material interface [31]. Cole–Cole plots (Fig. 2F and Figs. S2 to S6) reveal that in this topological insulator nanosheet composite, dielectric behavior is dominated by relaxation loss at low loading levels, whereas conduction loss dominates at high loading levels. There is a significant jump in both $\varepsilon^{\prime }$ and $\varepsilon^{\prime \prime }$ that is observed between 80 and 85 wt % in Fig. 2G and H. The jump arises from 2 synergistic mechanisms. First, Bi_2_Te_3_ nanosheets can be used as microcapacitors because of their surface-state conductivity and bulk phase insulation, and a large number of microcapacitors are combined to remarkable improve their dielectric polarization. Second, high-density heterogeneous interfaces between nanosheets and the paraffin matrix trigger Maxwell–Wagner polarization, which further augments $\varepsilon^{\prime }.$ The surge in $\varepsilon^{\prime \prime }$ originates from percolative conductive networks formed by nanosheets at high concentrations [32]. Crucially, suppressed conductive pathways within the insulating bulk phase maintain ε″ below ε′, avoiding dielectric runaway. Figure S7 reveals significant density of state peaks near the Fermi level (0 eV) for Bi-p orbitals (gray line) and Te-p orbitals (purple line), indicating that p-orbital hybridization between these states jointly dominates the surface conductive behavior. Additionally, as a nonmagnetic material [33], $M_{\eta }$ decreases with increasing filling ratio of Bi_2_Te_3_ nanosheets (Fig. 2D), which demonstrates deteriorated impedance matching between high filling ratio of Bi_2_Te_3_ nanosheets and air. α of all samples decreases with increasing frequency and escalates with increasing filling ratio of Bi_2_Te_3_ nanosheets (Fig. 2E). This suggests that all samples have strong microwave attenuation capability at high frequency. Moderate and well-balanced permittivity covering 2- to 18-GHz range is attributed to the unique charge transport properties of Bi_2_Te_3_ confined by Dirac surface states (Fig. 2I). These surface states are a unique feature of 3-dimensional topological insulators. They are characterized by massless Dirac fermions and manifest as single, nondegenerate Dirac cones. The cones exhibit linear dispersion relations, whose Dirac points are protected by time-reversal symmetry [34].

**Fig. 3.**
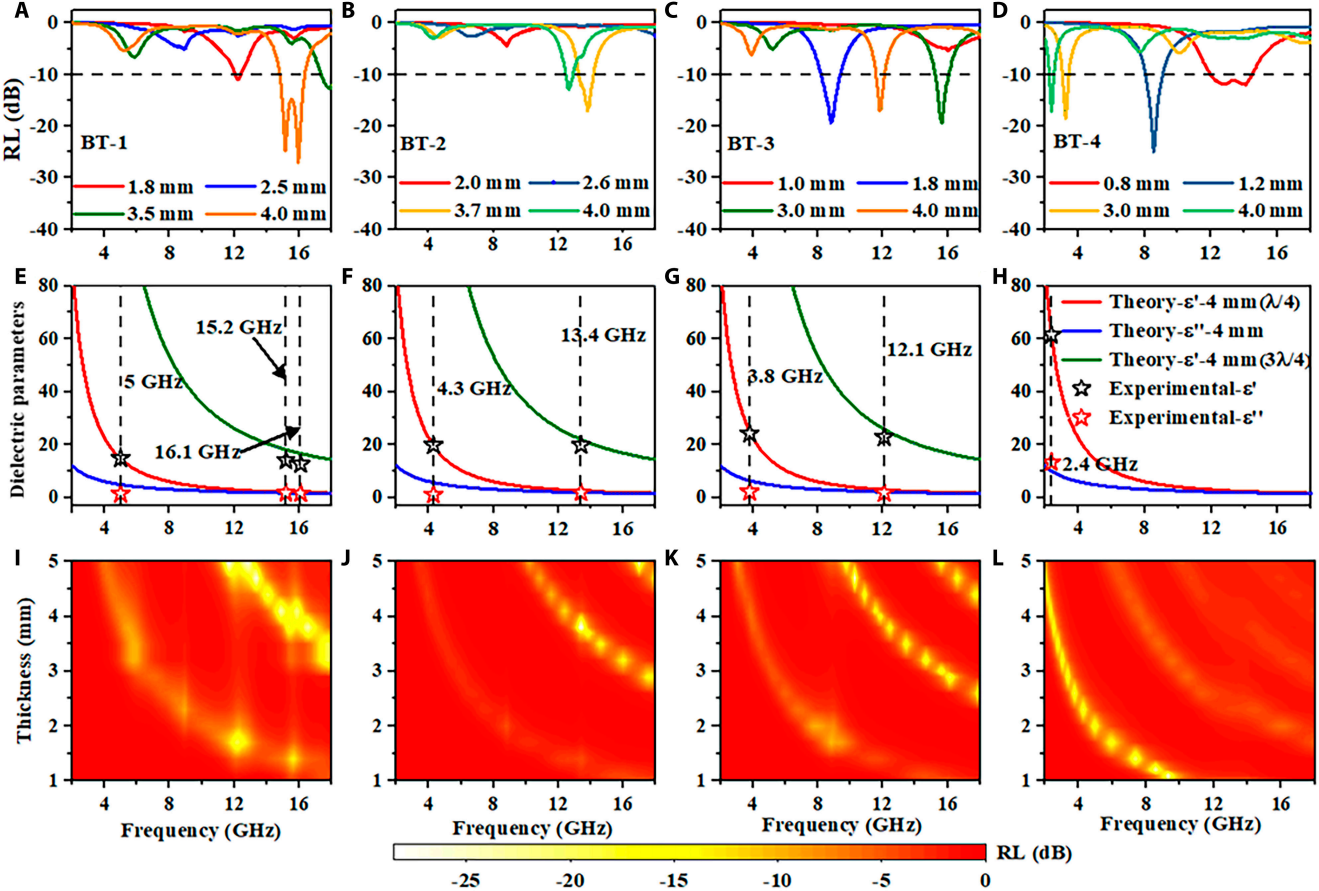
Frequency-dependent RL of (A) BT-1, (B) BT-2, (C) BT-3, and (D) BT-4 materials at different thicknesses. Comparison of experimental $\;\varepsilon^{\prime }$ and $\varepsilon^{\prime \prime }\;$of (E) BT-1, (F) BT-2, (G) BT-3, and (H) BT-4 materials with ideal dispersion curves of $\varepsilon^{\prime }$ and $\varepsilon^{\prime \prime }$at 4 mm. Contour map of RL of (I) BT-1, (J) BT-2, (K) BT-3, and (L) BT-4 materials.

Figure 3A to D and Fig. S8 show reflection loss (RL) curves of Bi_2_Te_3_ nanosheet composites. Here, the BT-80 wt % material achieves a strong RL close to 20 dB at 2.4 GHz with a thickness of 4 mm. It achieves an ultra-thin effective absorption electrical thickness of 0.032—defined as the ratio of physical thickness to resonance wavelength—substantially lower than state-of-the-art microwave absorbers reported to date (Table 1). The low electrical thickness not only enables effective absorption at reduced physical thickness but also facilitates the development of thinner multilayer absorbing structures and advances the miniaturization of microwave attenuators. As the thickness increases, the absorption peak shifts to lower frequency, which follows the quarter-wavelength resonance theory. Moreover, the absorption peak also shifts to lower frequency as the filling ratio of Bi_2_Te_3_ nanosheet increases at the same thickness. However, due to the mismatch between $\varepsilon^{\prime }$ and $\varepsilon^{\prime \prime }$, BT-5 exhibits inferior microwave absorbing performance compared to other materials. That is, the increase in $\varepsilon^{\prime }\;$ leads to an enhancement in refractive index, thereby satisfying the resonance conditions at lower frequencies at the same thickness (Figs. S9 to S12). Figure 3E to H shows the comparison of experimental dielectric parameters with the frequency dispersion curve of $\varepsilon^{\prime }$ and $\varepsilon^{\prime \prime }$ at 4 mm for ideal absorber. The dielectric dispersion curves of the ideal absorber exhibit a rapid decrease with increasing frequency [8]. When the experimental dielectric parameters fall on the theoretical dielectric curve, it indicates excellent absorption performance at the frequency. Conversely, when the parameters deviate from the theoretical curve, inferior absorption performance will be observed at the frequency. For example, the experimental $\varepsilon^{\prime }$ at 5 GHz falls on the theoretical curve of $\varepsilon^{\prime }$ for ^1^/_4_-wavelength resonance (Fig. 3E), so an absorption peak is observed at 5 GHz in Fig. 3A. But the experimental $\varepsilon^{\prime \prime }$ at 5 GHz deviates from the theoretical curve of $\varepsilon^{\prime \prime }$, resulting in inferior microwave absorption performance (<−10 dB) in Fig. 3A. The peaks at 15.2 and 16.1 GHz (Fig. 3A) are dominated by ^3^/_4_-wavelength resonance (Fig. 3E). The experimental $\varepsilon^{\prime }$ and $\varepsilon^{\prime \prime }$ at 2.4 GHz both fall on the theoretical curves (Fig. 3H), so a strong absorption peak is observed at 2.4 GHz in Fig. 3D. The input impedance matching coefficients $M_{z}$ [35] of Bi_2_Te_3_ nanosheet composites (Figs. S13 to S16) also confirm the above view. Figure 3I to L shows contour map of RL of Bi_2_Te_3_ nanosheet composites at different thicknesses with different filling ratios. Multiple absorption peaks are observed at the same thickness and frequency, which is in favor of broadband microwave absorption design.

**Table 1.** The electrical-thickness comparison of Bi_2_Te_3_ with other microwave absorbing materials in the literatures

Absorbers	Thickness (mm)	Frequency (GHz)	$\frac{d}{\lambda }$	Reference
FeNi/NC	5.5	3.8	0.07	[1]
Carbon/SiC	3.0	8.5	0.086	[3]
Co@NC	5.0	4.1	0.068	[5]
SCF@TiO_2_	4.0	3.92	0.52	[8]
SiCN/M	4.0	3.56	0.048	[12]
FeSiAl	5.1	3.47	0.060	[41]
BN@FeCo	5.0	3.1	0.052	[42]
CFC-LC	5.0	2.0	0.033	[43]
Fe-N-C/ZrO_2_	5.0	3.9	0.065	[44]
BN@CoC@C	5.0	3.8	0.063	[45]
CNF@Co/C	4.5	7.0	0.105	[46]
Bi_2_Te_3_	4.0	2.4	0.032	This work

**Fig. 4.**
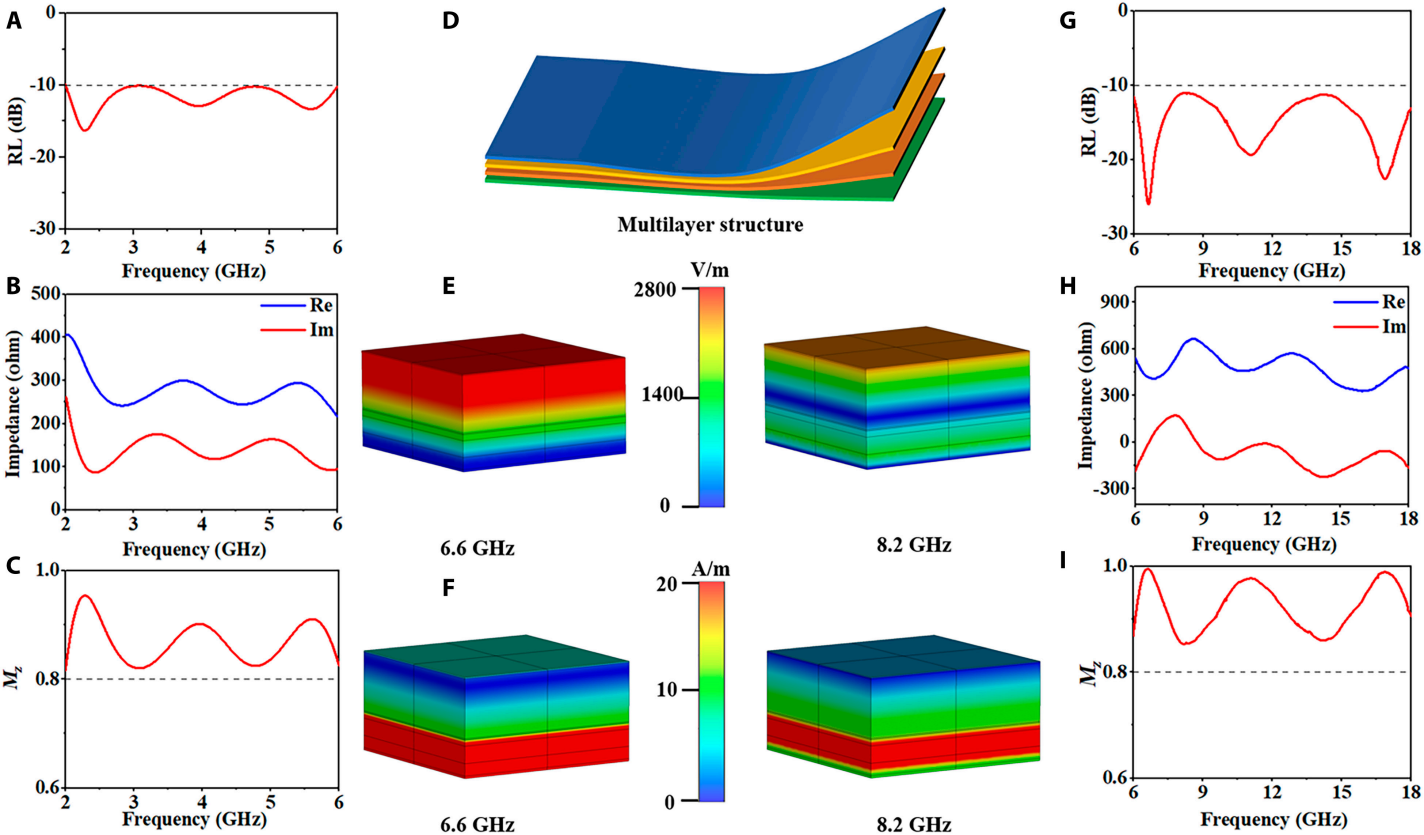
(A) RL, (B) input impedance, and (C) $M_{z}$ of 8-layer structure. (D) Schematic of multilayer structure, (E) electric field distributions at 6.6 and 8.2 GHz, and (F) magnetic field distributions at 6.6 and 8.2 GHz. (G) RL, (H) input impedance, and (I) $M_{z}$ of 7-layer structure.

In order to obtain broadband microwave absorption, multilayer architectures featuring a gradient distribution of $\varepsilon^{\prime }$ are employed to facilitate quarter-wavelength resonances at different frequencies, thereby enabling multi-frequency impedance matching. Two multilayer architectures (Figs. S17 and S18) were fabricated using high-permittivity Bi_2_Te_3_ nanosheet composites (with 5 distinct filling ratios) and low-permittivity rubber. The layer stacking sequence and thickness distribution were optimized via an intelligent algorithm. Leveraging the strong frequency dispersion of BT-85 wt % material, 8-layer structure absorber (Table S1) achieves broadband microwave absorption covering 2 to 6 GHz at thickness of 9.8 mm (Fig. 4A). The 7-layer structure absorber (Table S2) realizes broadband microwave absorption covering 6 to 18 GHz at thickness of 4.1 mm (Fig. 4G). Figure 4B and H shows the real and imaginary parts of the input impedances of the multilayer absorbers, respectively. The corresponding $M_{z}$ can be calculated using Eq. (S1), as shown in Fig. 4C and I. $M_{z}$ values of the 2 multilayer structures are both larger than 0.8, so RL values are both less than −10 dB. When $M_{z}$ = 0.99 at 6.6 GHz (Fig. 4I), the corresponding input impedance real part is ~377 Ω and the imaginary part is ~0 Ω(Fig. 4H), indicating excellent impedance matching with air at this frequency point. The broadband microwave absorption of absorbers results from the multi-frequency ^1^/_4_-wavelength resonances induced by multilayer stacking. Figure 4E and F demonstrates the electric and magnetic field distributions of 7-layer structure at 6.6 and 8.2 GHz, respectively. A progressive attenuation of electric field intensity (Fig. 4E) is observed from the surface of absorber to the metal backplate, corresponding to the electromagnetic wave phase transition from peak to null, which is a typical character of ^1^/_4_-wavelength resonance at 6.6 GHz. The electromagnetic field distribution shows a slight deviation from the quarter-wavelength condition at 8.2 GHz, confirming the lower absorption intensity at this frequency compared to that at 6.6 GHz. It is noteworthy that the BT-85 wt % material plays a dominant role in the coexistence of multiple ^1^/_4_-wavelength resonances at different frequencies due to the strong dielectric dispersion.

## Conclusion

In summary, Bi_2_Te_3_ nanosheets were prepared via a solvothermal method. The materials exhibit adjustable high permittivity and appropriate dielectric loss due to the unique characteristics of bulk insulating and surface conducting. The permittivity of Bi_2_Te_3_ nanosheet composites can be flexibly tuned from 5 to 282. When the permittivity is up to 282, dielectric loss tangent is still less than 0.81, which can facilitate broadband design for low-frequency microwave absorption. The Bi_2_Te_3_ nanosheet composite achieves a minimum RL at a thickness of 0.032*λ* at 2.4 GHz. Leveraging the strong dielectric dispersion of Bi_2_Te_3_ nanosheets, 2 multilayer patches with 5 distinct filler loadings were designed. The multilayer structures achieved broadband effective absorption covering 2- to 6-GHz range and 6- to 18-GHz range, respectively. This study proposed a material with ultra-high adjustable permittivity, offering a feasible approach to realize broadband low-frequency microwave absorption.

## Methods

### Preparation of Bi_2_Te_3_ nanosheets

Firstly, bismuth nitrate [Bi(NO_3_)_3_, 2 mM, 99.9%, Aladdin], tellurium dioxide (TeO_2_, 3 mM, 99.9%, Aladdin), sodium hydroxide (NaOH, 16 mM, 98%, Aladdin), and varying amounts of polyvinyl pyrrolidone (PVP, 99%, Aladdin) were acquired separately. Then, ethylene glycol monomethyl ether (36 ml) was measured and transferred to a glass beaker. PVP was fully dissolved in ethylene glycol monomethyl ether using a digital ultrasonic cleaner until a homogeneous transparent solution was obtained. Under continuous magnetic stirring, the bismuth nitrate and tellurium dioxide powders (molar ratio 2:3) were gradually added into the above solution until complete dissolution was achieved. Subsequently, sodium hydroxide was dissolved in 4 ml of deionized water under mechanical agitation to prepare a 4 M aqueous solution. All prepared solutions were subject to vigorous magnetic stirring at room temperature for 0.5 h. Then, the homogeneous mixture was transferred into a 50-ml polytetrafluoroethylene (PTFE)-lined autoclave. After sealing and heating at 180 °C for 24 h, the synthesized product was rinsed 3 times with acetone via vacuum filtration, followed by vacuum drying at 70 °C for 2 h, yielding Bi_2_Te_3_ nanosheets.

### Characterizations and theory

The morphological and microstructural features of Bi_2_Te_3_ were characterized via SEM (Hitachi S4800), TEM (FEI Tecnai G2 F30), and HRTEM (FEI Tecnai G2 F30). The SAED of the Bi_2_Te_3_ nanostructures was analyzed using a JEOL JEM-2100F microscope. The elemental distribution was characterized using energy-dispersive x-ray spectroscopy (EDS) mapping. The crystal structure of Bi_2_Te_3_ nanosheets was analyzed by XRD in the range of 15° to 85° using Rigaku Ultima IV (Cu Kα). The elemental composition of Bi_2_Te_3_ nanosheets was analyzed using XPS (Thermo Escalab 250XI). Bi_2_Te_3_ nanosheets were blended with paraffin wax at 5 mass ratios as 50:50, 60:40, 70:30, 80:20, and 85:15, which were denoted as BT-1, BT-2, BT-3, BT-4, and BT-5, respectively. Annular samples of Bi_2_Te_3_ nanosheets with an outer diameter of 6.93 mm, an inner diameter of 3.06 mm, and a thickness of 1.98 mm were fabricated, and their complex permeability and complex permittivity were measured covering 2 to 18 GHz by a vector network analyzer (Keysight E5071C). The 8-layer structure absorber and the 7-layer structure absorber were simulated using the microwave simulation software (CST) version 2014.

The ^1^/_4_-wavelength resonance equation explains the relationship between the material thickness and the absorption peak frequency, and the equation [8] is as follows:

d=2m−14λ=2m−1c4nfm=123… (1)

where *d* represents the thickness of the composite material, *f* is the corresponding frequency, *c* is the speed of light in a vacuum, *m* is natural integer (*m* = 1, 2, 3, …), and *n* = Reμrεr is the refractive index of the composite material. RL is a parameter used to evaluate the microwave absorption properties of materials and can be calculated using the following equations [36–38]:

RLdB=20logZin−Z0Zin+Z0 (2)

$$Z_{\text{in}}=Z_{0}\sqrt{\frac{\mu_{r}}{\varepsilon_{r}}}\tanh\left[j\left(\frac{2\pi fd}{c}\right)\sqrt{\mu_{r}\varepsilon_{r}}\right]$$ (3)

where *Z_in_* is the input impedance of the absorber and *d* is the thickness of the absorber. The attenuation constant is a parameter in determining the wave absorption characteristics of materials and can be calculated using the following equation [39]:

α=2πfc×μ″ε″−μ′ε′+μ″ε″−μ′ε′2+μ′ε″+μ″ε′2 (4)

where *f* and *c* represent the frequency and the speed of light, respectively. The wave impedance matching coefficient $M_{\eta }$ serves as a critical parameter for evaluating the impedance matching characteristics of materials under the condition of infinite thickness. It can be calculated using the following equation [40]:

Mη=2η′∣η∣2+1 (5)

where $\varepsilon_{r}=\varepsilon^{\prime }-j\varepsilon^{\prime \prime }$ and $\;\mu_{r}=\mu^{\prime }-\mu^{\prime \prime }$ represent the relative complex permittivity and relative complex permeability, respectively. Relative wave impedance $\eta =\sqrt{\mu_{r}/\varepsilon_{r}},\eta^{\prime }\;$ is the real part of relative wave impedance η. When $M_{\eta }$ = 1, the material and air can achieve the ideal impedance matching. Therefore, when the $\;M_{\eta }$ value approaches 1, the tested sample exhibits better microwave absorption performance. For nonmagnetic materials, the dielectric dispersion relation of an ideal absorber is given by Eqs. (6) and (7) [9].

εr′=c28f2d21+Tan2δ21+1+Tan2δ (6)

$$\varepsilon_{r}^{\prime \prime }=\frac{c}{\pi fd}$$ (7)

where Tanδ is the tangent of the loss angle and *d* is the thickness of the absorber. When the $\varepsilon_{r}^{\prime }$ of a material at a specific frequency satisfies Eq. (6) (the ideal dielectric parameter), it indicates that the quarter-wavelength resonance can be realized at this frequency. If $\varepsilon_{r}^{\prime }$ and $\varepsilon_{r}^{\prime \prime }$ simultaneously satisfy Eqs. (6) and (7) at a specific frequency, respectively, the normalized input impedance *Z_in_*/*Z*_0_ equals 1 at the resonant thickness. That is, the absorber achieves perfect absorption at the resonant thickness.

## Data Availability

All data are available in the main text or the Supplementary Materials. Source data are available from the corresponding author upon reasonable request.
