# Supplementary material for: Ultrahigh Permittivity of Surface-State-Dominated Bi2Te3 Nanosheets for Low-Frequency Microwave Absorption
Source: Research (Wash D C). 2025 Oct 22;8:0886. doi: 10.34133/research.0886 (PMC12541145; doi:10.34133/research.0886)
Supplement: Supplementary 1 — Supplementary Text Figs. S1 to S19 Tables S1 and S2 [file research.0886.f1.docx]

**Ultrahigh Permittivity of Surface-State-Dominated Bi_2_Te_3_ Nanosheets for Low-Frequency Microwave Absorption**

Dengchen Li^1,2^, Qiji Ma^3^, Chengyou Lin^3^, Chen-Ming Liang^1^, Ling Huang^2^, Yuhang Qi^2^, Jianhua Li^2^, Pei-Yan Zhao^1^, Zhi-Ling Hou^4^, Dongfeng Zhang^1*^, Guang-Sheng Wang^1*^

*^1^ School of Chemistry, Beihang University, Beijing 100191, China*

*^2^ CH UAV Science & Technology Co., Ltd, Taizhou 318000, China*

*^3^* *College of Mathematics and Physics, Beijing University of Chemical Technology, Beijing 100029, China*

*^4^ School of Physics and Optoelectronic Engineering, Beijing University of Technology, Beijing 100124, China*

*Corresponding authors: Dongfeng Zhang, dfzhang@buaa.edu.cn; Guang-Sheng Wang, wanggsh@buaa.edu.cn

For a microwave absorbing material of a given thickness, the microwave absorption performance is uniquely determined by the input impedance matching:

$$\begin{aligned} M_{z}=\frac{2Z_{\mathrm{in}}^{'}}{|Z_{in}|^{2}+1}\#\left（ S1 \right） \end{aligned}$$

where$Z_{\mathrm{in}}^{'}$ is the real part of the normalized input impedance, while$Z_{in}$ is the normalized input impedance. When the normalized impedance coefficient ($M_{z}$) approaches unity, the corresponding absorption peak intensity is significantly enhanced. Perfect impedance matching ($M_{z}$= 1) is achieved when this condition is satisfied, resulting in optimal microwave absorption performance.

For non-magnetic materials, When the $\varepsilon_{r}^{'}$ of a material at a specific frequency satisfies Formula (S2) (the ideal dielectric parameter), it indicates that this frequency point achieves three-quarter wavelength resonance.

$$\begin{aligned} \varepsilon_{r}^{'}=\frac{9c^{2}}{8f^{2}d^{2}}\frac{1+\frac{Tan^{2}\delta}{2}}{1+\sqrt{1+Tan^{2}\delta}}\# \end{aligned}\left（ S2 \right）$$

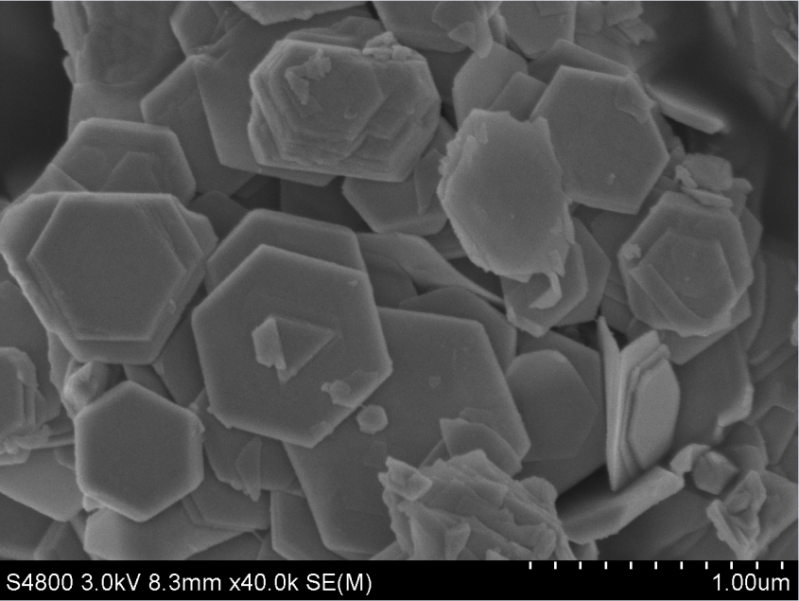


Figure S1 SEM image of Bi_2_Te_3_ nanosheets


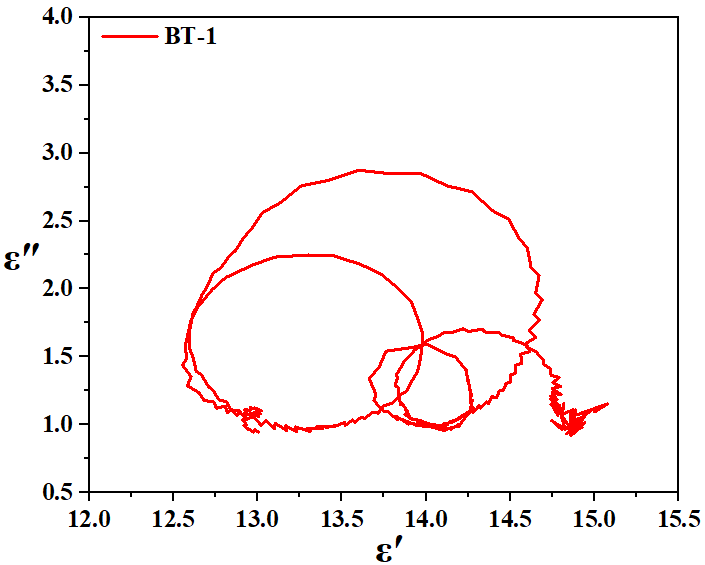


Figure S2 Cole-Cole curve of BT-1


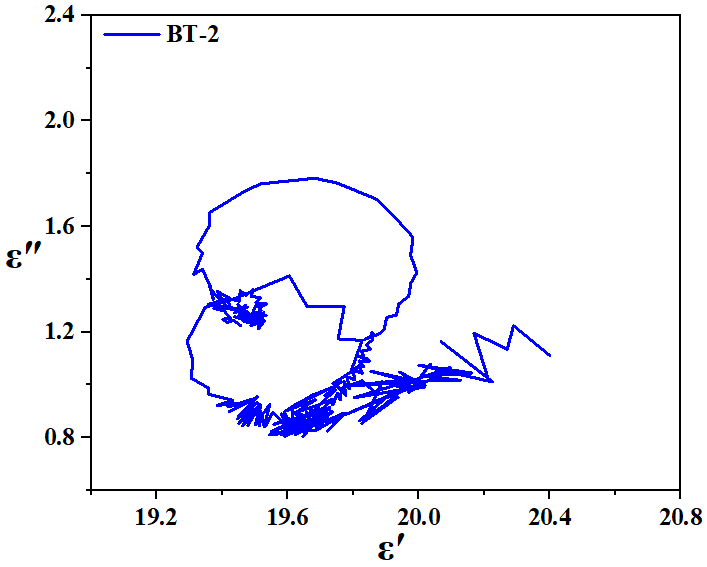


Figure S3 Cole-Cole curve of BT-2


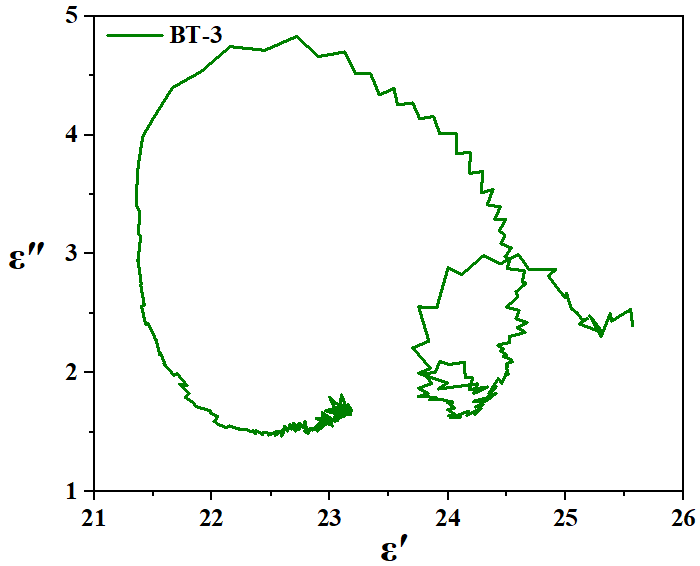


Figure S4 Cole-Cole curve of BT-3


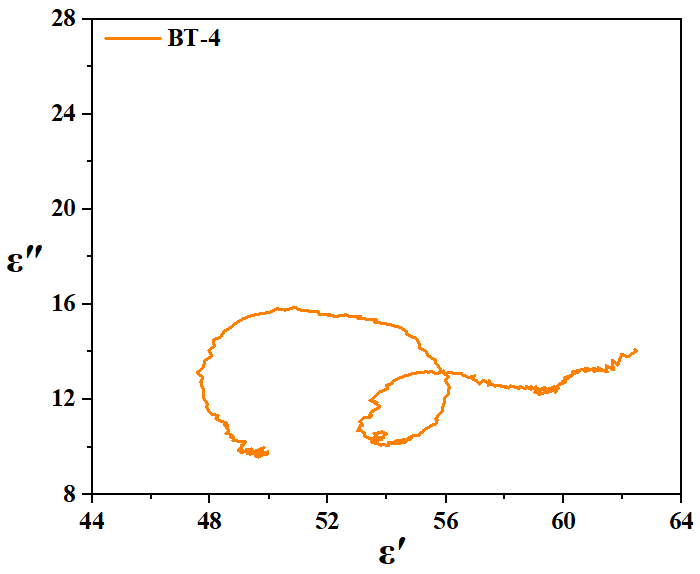


Figure S5 Cole-Cole curve of BT-4


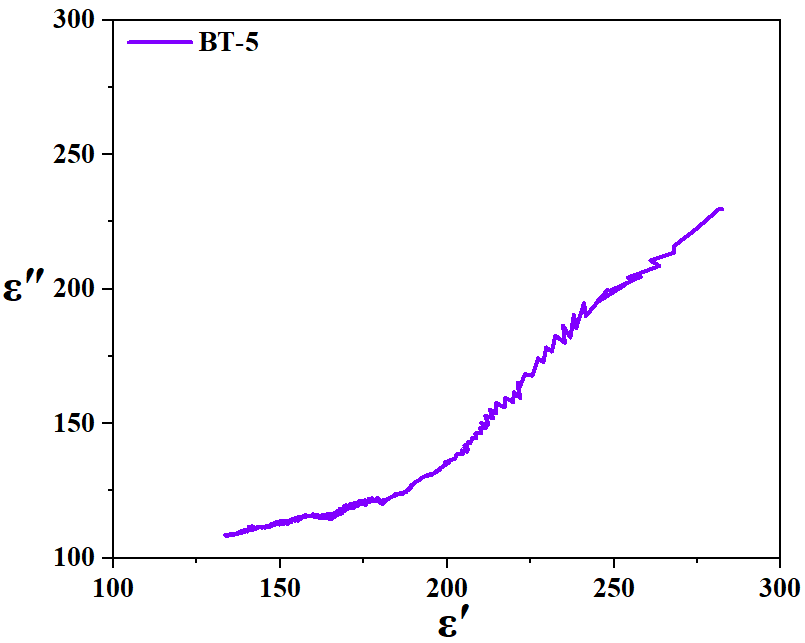


Figure S6 Cole-Cole curve of BT-5

First-principles calculations were performed using the Vienna ab initio Simulation Package (VASP) based on density functional theory (DFT). The projector augmented wave (PAW) method was employed to describe ion-electron interactions, while the exchange-correlation functional was treated within the Perdew-Burke-Ernzerhof (PBE) generalized gradient approximation (GGA). Computational parameters include a 400 eV plane-wave energy cutoff, a 20 Å vacuum space along the z-direction, and convergence criteria of 0.001 eV/Å for atomic forces and 1×10^-6^ eV for total energy.


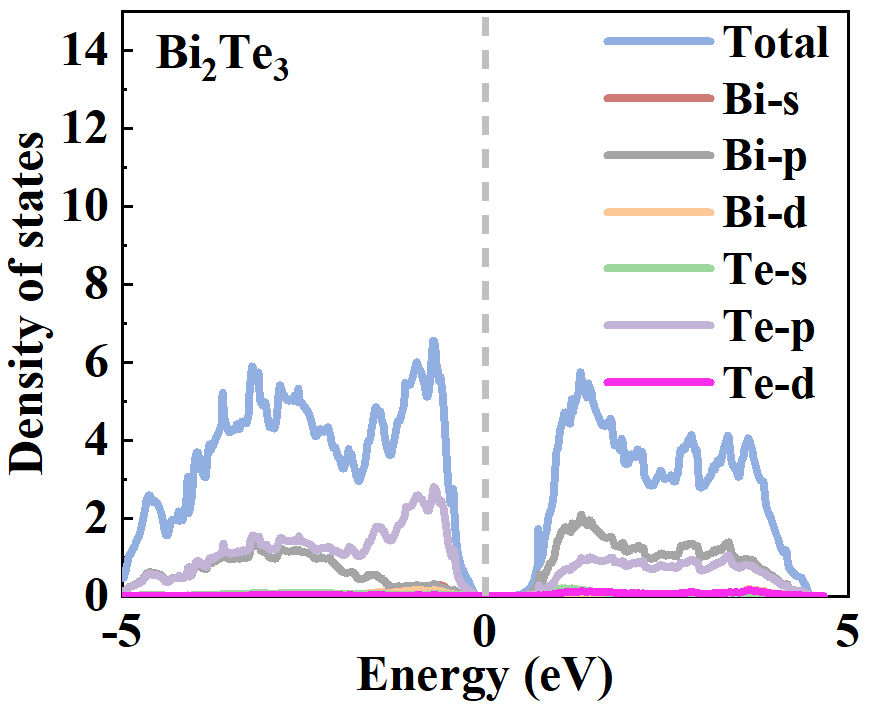


Figure S7 Density of states of the Bi_2_Te_3_


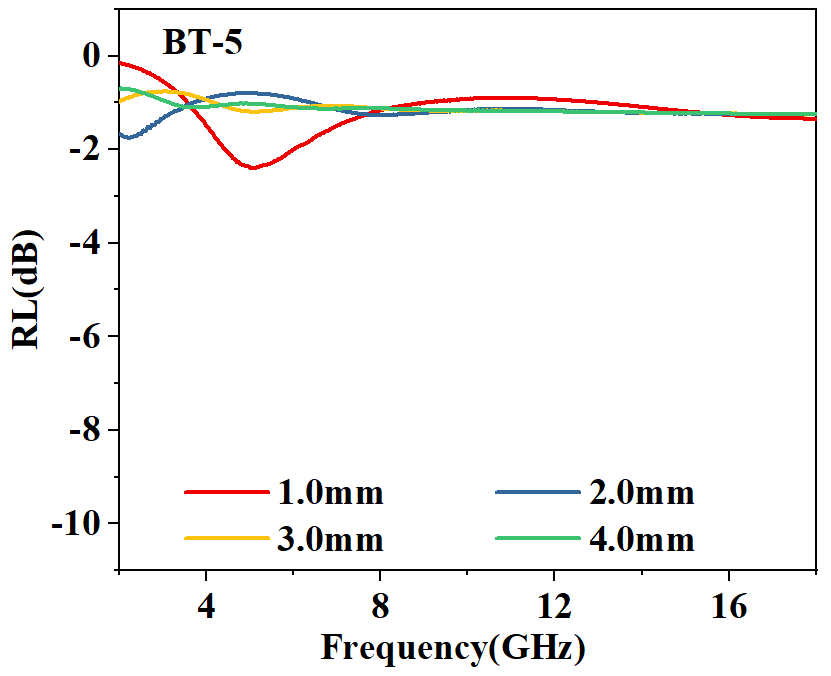


Figure S8 Frequency-dependent RL of BT-5 materials at different thicknesses.


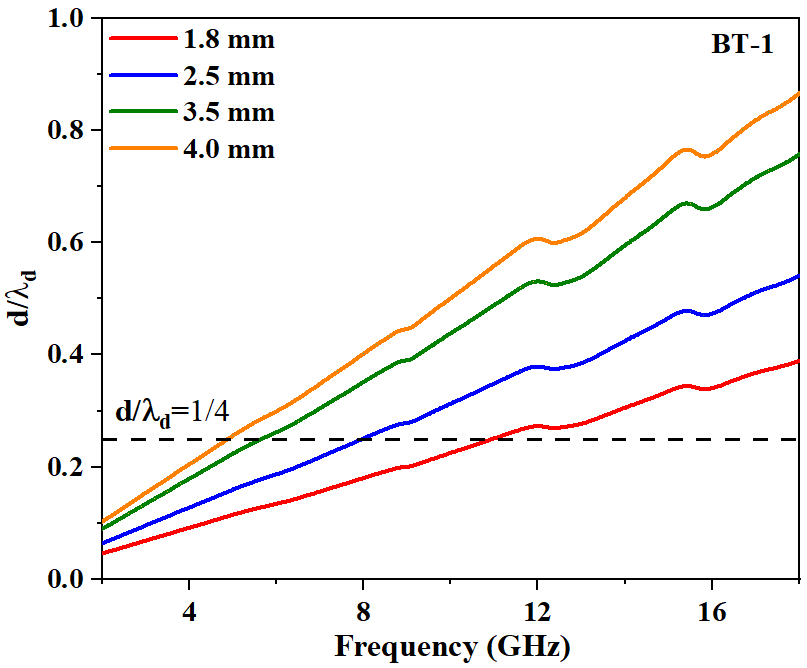


Figure S9 Frequency-dependent d/$\lambda_{d}$of BT-1


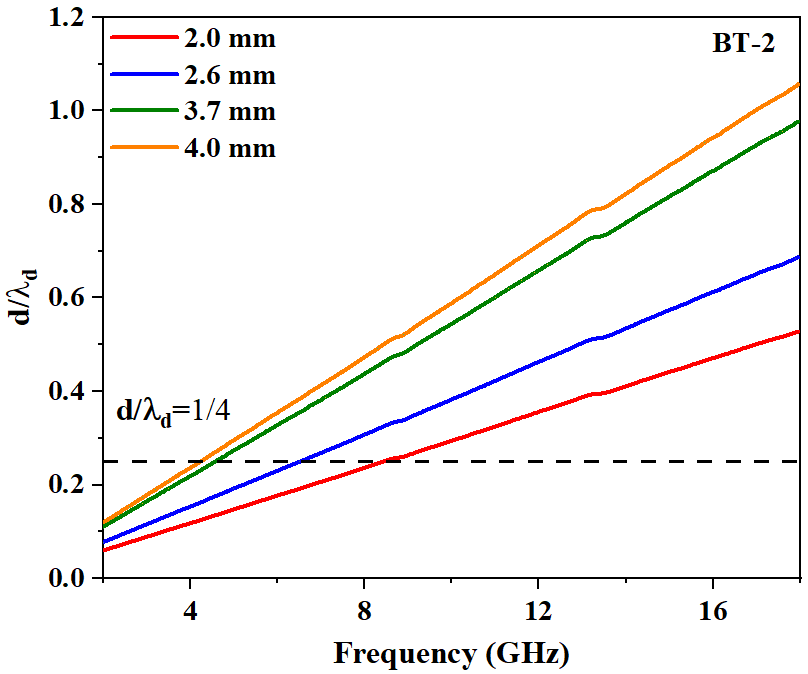


Figure S10 Frequency-dependent d/$\lambda_{d}$of BT-2


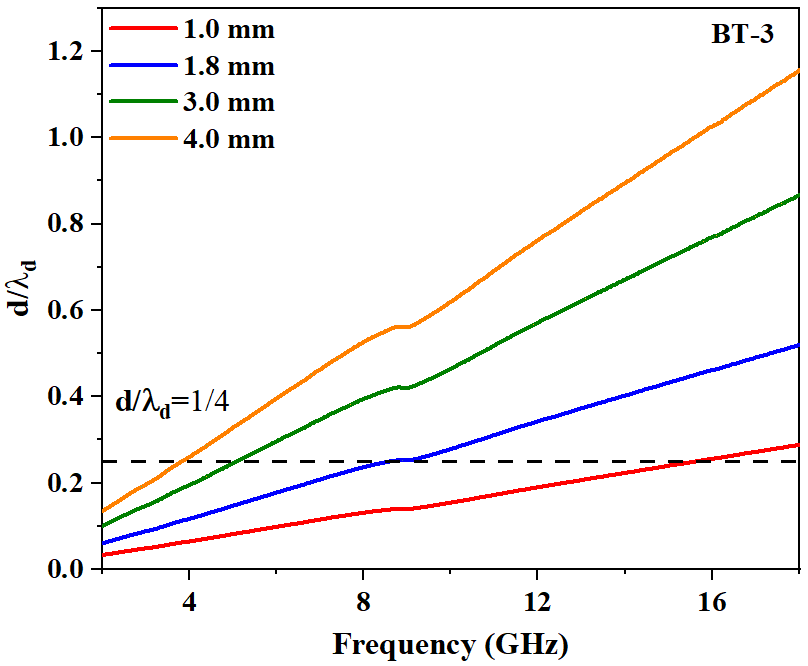


Figure S11 Frequency-dependent d/$\lambda_{d}$of BT-3


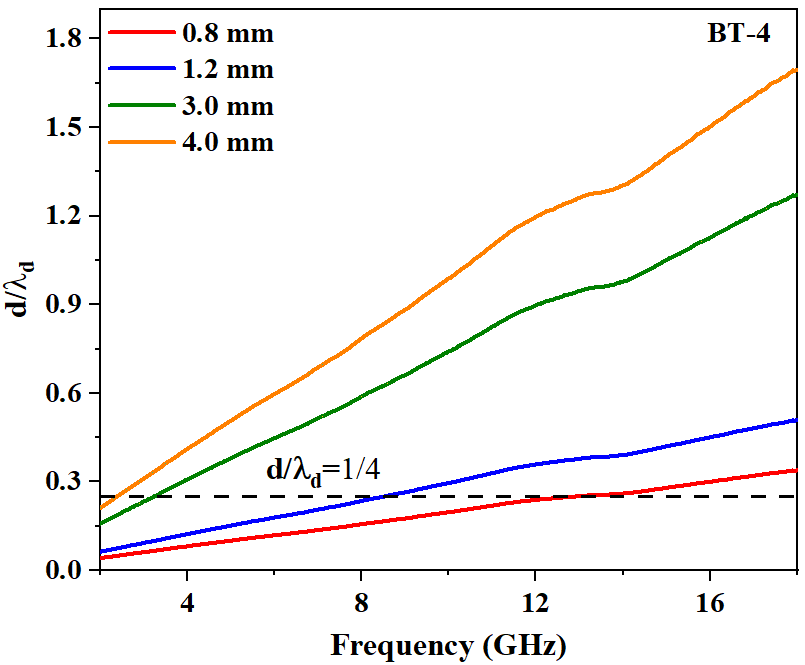


Figure S12 Frequency-dependent d/$\lambda_{d}$of BT-4


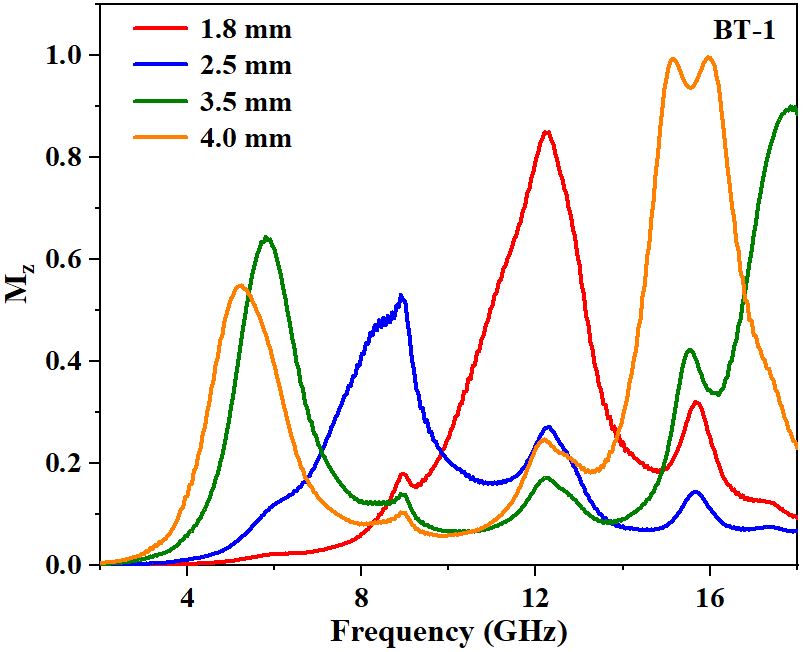


Figure S13$M_{z}$of BT-1


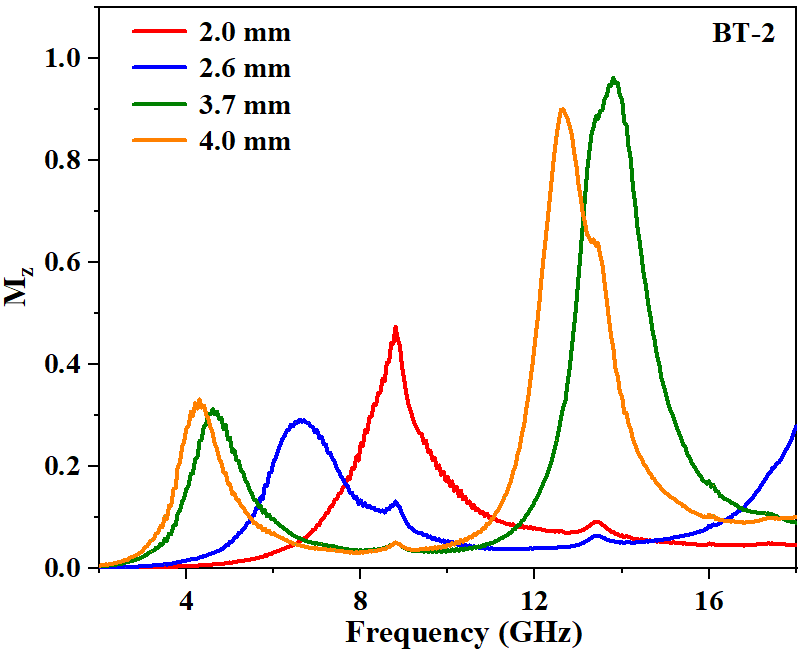


Figure S14$M_{z}$of BT-2


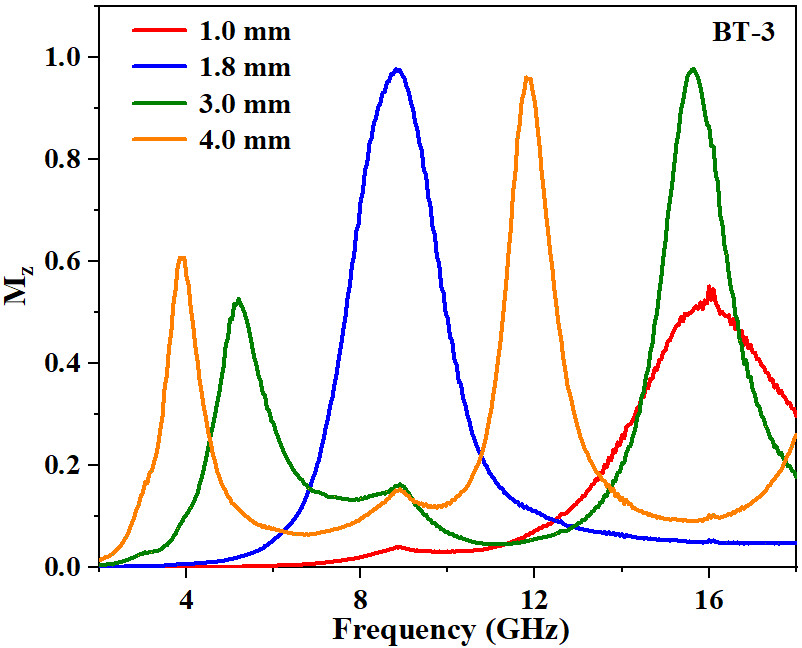


Figure S15$M_{z}$of BT-3


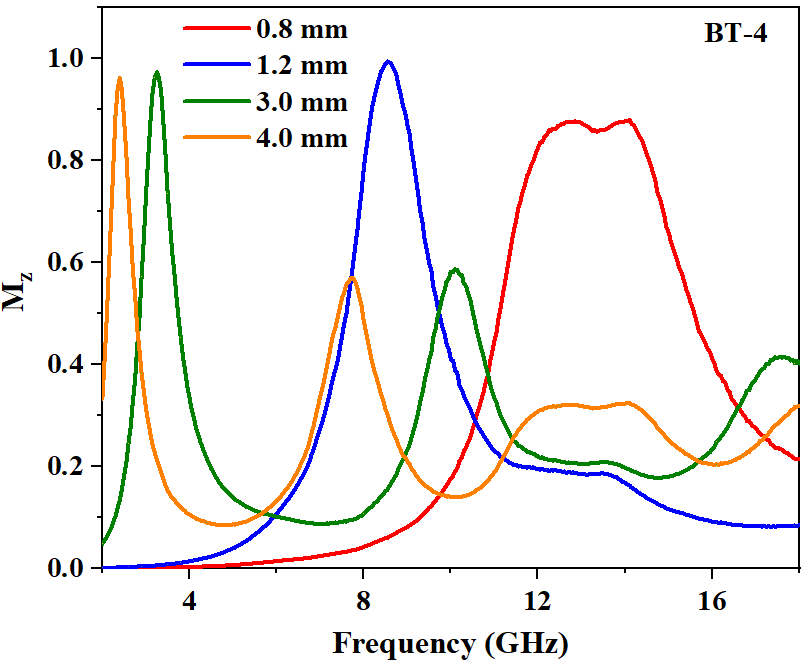


Figure S16$M_{z}$of BT-4


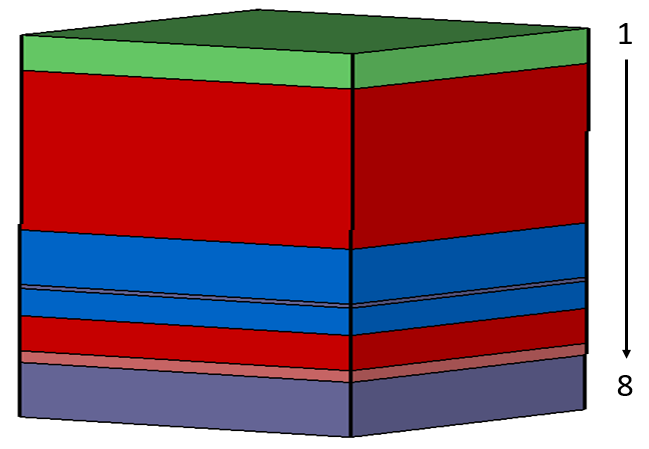


Figure S17 Eight-layer structure absorber.

Eight-layer structure absorber with the sequential composition and corresponding thickness parameters from top to bottom as follows:

Table S1 Layer material composition and thickness

| Layer | Composition | Thickness (mm) |
| --- | --- | --- |
| 1 | BT-2 composite | 0.9 |
| 2 | Pure rubber | 4.1 |
| 3 | BT-3 composite | 1.4 |
| 4 | BT-5 composite | 0.1 |
| 5 | BT-3 composite | 0.7 |
| 6 | Pure rubber | 0.9 |
| 7 | BT-1 composite | 0.3 |
| 8 | BT-5 composite | 1.4 |


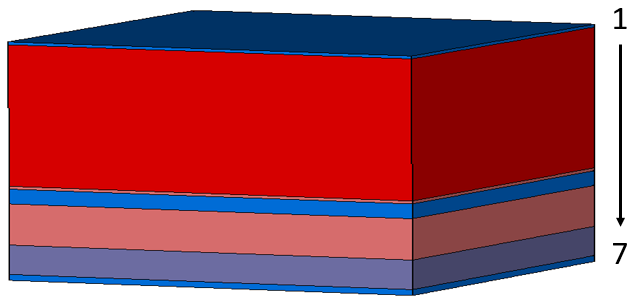


Figure S18 Seven-layer structure absorber.

Seven-layer structure absorber with the sequential composition and corresponding thickness parameters from top to bottom as follows:

Table S2 Layer material composition and thickness

| Layer | Composition | Thickness (mm) |
| --- | --- | --- |
| 1 | BT-4 composite | 0.05 |
| 2 | Pure rubber | 2.4 |
| 3 | BT-1 composite | 0.05 |
| 4 | BT-4 composite | 0.3 |
| 5 | BT-1 composite | 0.7 |
| 6 | BT-5 composite | 0.5 |
| 7 | BT-4 composite | 0.1 |


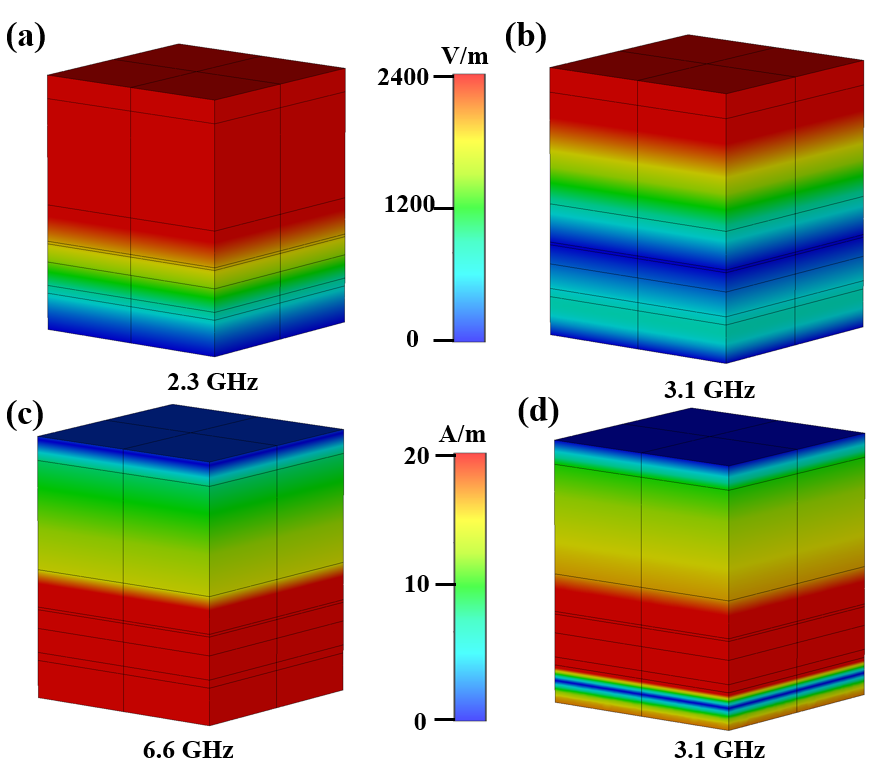


Figure S19 (a) and (b) Electric field distribution, (c) and (d) Magnetic field distribution of eight-layer structure absorber.
